# Impact of pulmonary exacerbations and lung function on generic health-related quality of life in patients with cystic fibrosis

**DOI:** 10.1186/s12955-016-0465-z

**Published:** 2016-04-21

**Authors:** Caitlyn T. Solem, Montserrat Vera-Llonch, Sizhu Liu, Marc Botteman, Brenda Castiglione

**Affiliations:** Pharmerit International, 4350 East West Hwy, Suite 430, Bethesda, MD 20814 USA; Vertex Pharmaceuticals, 50 Northern Ave, Boston, MA 02210 USA

**Keywords:** Cystic fibrosis, EQ-5D, Lung function, Pulmonary exacerbation

## Abstract

**Background:**

The analysis aimed to examine the impact of pulmonary exacerbations (PEs) and lung function on generic measures of HRQL in patients with cystic fibrosis (CF) using trial-based data.

**Methods:**

In a 48-week randomized, placebo-controlled study of ivacaftor in patients ≥12 years with CF and a *G551D*-CFTR mutation the relationship between PEs, PE-related hospitalizations and percent predicted forced expiratory volume in one second (ppFEV_1_) with EQ-5D measures (index and visual analog scale [VAS]) was examined in post-hoc analyses. Multivariate mixed-effects models were employed to describe the association of PEs, PE-related hospitalizations, and ppFEV_1_ on EQ-5D measures.

**Results:**

One hundred sixty one patients (age: mean 25.5 [SD 9.5] years; baseline ppFEV_1_: 63.6 [16.4]) contributed 1,214 observations (ppFEV_1_: no lung dysfunction [*n* = 157], mild [*n* = 419], moderate [*n* = 572], severe [*n* = 66]). Problems were most frequently reported on pain/discomfort, anxiety/depression, and usual activities EQ-5D items. The mean (SE) EQ-5D index nominally decreased (worsened) with worsening severity of lung dysfunction (*P* = 0.070): 0.931 (0.023); mild: 0.923 (0.021); moderate: 0.904 (0.018); severe: 0.870 (0.020). 146 PEs were experienced by 72 patients, including 52 PEs (35.6 %) that required hospitalization. Mean EQ-5D index and VAS scores were lowest (worst) within 1 week (before or after PE start) for PEs requiring hospitalization. Pulmonary exacerbations, PE-related hospitalizations, and ppFEV_1_ were significant predictors of EQ-5D index and VAS.

**Conclusions:**

In a clinical study of patients with CF (≥12 years of age and a *G551D*-CFTR mutation), PEs, primarily those requiring hospitalization, were associated with low EQ-5D index and VAS scores. The impact of ppFEV_1_ was relatively smaller. Reducing PEs, in particular those requiring hospitalization, would likely improve HRQL among these patients.

**Trial registration:**

ClinicalTrials.gov, NCT00909532; URL: clinicaltrials.gov, May 26, 2009

**Electronic supplementary material:**

The online version of this article (doi:10.1186/s12955-016-0465-z) contains supplementary material, which is available to authorized users.

## Background

Health-related quality of life (HRQL) is an increasingly important multi-dimensional clinical outcome assessment which provides insights into the patient’s experience of disease burden and the effects of medical interventions. Measures of HRQL are patient-reported and can be generic or disease-specific [1]. Disease-specific measures may be more sensitive to some of the symptoms experienced by patients but generic measures permit uniform comparisons across medical conditions and as such are necessary to aid decision making in evaluating the value of new treatments. As the landscape of therapies for cystic fibrosis (CF) expands, it is important to characterize the relationship between clinical and physiologic measures of disease and generic measures of HRQL.

Cystic fibrosis is an inherited, rare autosomal recessive disease that results in chronically debilitating morbidities and high premature mortality [2]. CF disease affects multiple organs in the body including the lung, pancreas, intestinal and biliary tracts, sweat glands and the reproductive system [3]. Patients with CF typically experience progressive loss of lung function ultimately resulting in respiratory failure and death [2]. A key characteristic affecting CF disease trajectory is the occurrence of pulmonary exacerbations (PEs), which require acute medical care and often hospitalization. Avoiding PEs is a foremost goal of CF treatment since exacerbation frequency is associated with lung function decline, greater likelihood of subsequent exacerbations and increased mortality [4]. Pulmonary exacerbations, particularly those that are severe, have been reported to impact HRQL [5–8]. Lung function as measured by forced expiratory volume (FEV_1_) is an outcome measure in clinical studies of CF therapies and has been shown to be related to patient’s survival and HRQL in cross-sectional and longitudinal studies [8, 9].

Research to date has reported mixed degrees of associations between several CF physiologic and clinical measures such as FEV_1_ and PEs, and generic and disease-specific HRQL measures (e.g. Cystic Fibrosis Questionnaire-Revised [CFQ-R] scores) [5–8] of CF symptoms and HRQL. Previous studies of patients with CF have reported utilities derived using time tradeoff [7, 10–12] and standard gamble [7, 13, 14] direct elicitation methods, and generic indirect measures including the health utilities index (HUI2 and HUI3) [7, 14, 15], and the EuroQol EQ-5D [6, 16, 17] with varying results. Within CF there has been evidence of potential ceiling effects of generic measures such as the EQ-5D and insensitivity particularly to FEV_1_. While some of these studies have independently assessed the impact of lung function and PEs on generic measures of HRQL in CF, no studies to date have analyzed both of these key disease elements within the same model. In order to better assess this, the aim of this analysis is to examine the impact of PEs and lung function on generic HRQL as assessed by the EQ-5D-3 L questionnaire in patients aged 12 years and older with CF and a *G551D* mutation on at least 1 *CFTR* allele using data from the STRIVE clinical trial [18].

## Methods

### Data source

This analysis used data from a 48-week, Phase 3, international, multicenter, randomized, double-blind, placebo-controlled study (STRIVE) which was designed to evaluate the efficacy and safety of ivacaftor in patients aged 12 years and older with CF who had a *G551D* mutation on at least 1 *CFTR* allele [18]. Patients were included if they had an FEV_1_ of 40 to 90 % (inclusive) of the predicted normal value for age-, gender-, and height-matched persons at screening (ppFEV_1_). As reported in Ramsey et al. [18], “subjects were excluded if they had other illnesses that confounded the study results; ongoing illness; a pulmonary exacerbation or changes in therapy (including antibiotics) for pulmonary disease within 4 weeks before first dose of study drug; abnormal liver function tests, defined as 3 or more LFT parameters >3 times the upper limit of normal; or abnormal renal function tests. Subjects were also excluded if they had a history of prolonged QT/QTc interval; history of solid organ or hematological transplantation; colonization with organisms associated with a more rapid decline in pulmonary status (e.g., *B. cenocepacia, B. dolosa*, and *M. abcessus*); concomitant use of any inhibitors or inducers of CYP3A4; or use of inhaled hypertonic saline treatment. Subjects were required to stop inhaled hypertonic saline treatment for at least 4 weeks prior to Day 1 (first dose of study drug)”. Study assessments were conducted at baseline, day 15, week 8, and every 8 weeks thereafter through 48 weeks.

### Study measures

#### Health-related quality of life

The EQ-5D-3 L [19] is a generic measure of HRQL that includes two components: a descriptive profile and a visual analogue scale (VAS). The descriptive profile includes five single-item dimensions (mobility, self-care, usual activities, pain/discomfort, and anxiety/depression), each with 3 levels of response (no problems, some problems, and extreme problems) that can be combined into a single score (index) which summarizes health status (i.e., utility) and is anchored at 0 (=death) and 1 (=perfect health). The index is calculated by an algorithm using patients’ responses to the EQ-5D descriptive profile and preference weights for different health states ascertained from the general population of a country (herein, using values for the United Kingdom [UK]) [20].

The EQ-5D VAS records the respondent’s self-rated health on a 20 cm vertical VAS with endpoints labeled “the best health you can imagine” and “the worst health you can imagine.” [19]. This information can be used as a quantitative measure of health status as rated by the individual respondents.

#### Lung function

In the STRIVE study, ppFEV_1_ was the primary outcome measure. In our analyses, we characterize lung dysfunction respectively as: (1) no lung dysfunction (ppFEV_1_ ≥ 90 %), mild (70 ≤ ppFEV_1_ < 90 %), moderate (40 ≤ ppFEV_1_ < 70 %), and severe (FEV_1_ < 40 %); (2) deciles of ppFEV_1_; and (3) ppFEV_1_ treated as a continuous variable in multivariate analyses. Note that while patients were required to have at least mild lung dysfunction at entry to the trial, it was possible for patients to improve and have observations with no lung dysfunction at later points within the trial.

#### Pulmonary exacerbations

In the clinical study, PEs were defined as a change in antibiotic therapy for ≥4 of 12 signs or pre-defined sino-pulmonary symptoms (change in sputum; new or increased hemoptysis; increased cough; increased dyspnea; malaise, fatigue, or lethargy; temperature above 38 °C; anorexia or weight loss; sinus pain or tenderness; change in sinus discharge; change in physical examination of the chest; decrease in pulmonary function by 10 %; radiographic changes indicative of pulmonary infection). PE start and end dates and information on whether PEs required hospitalization or not was also collected.

### Statistical analysis

EQ-5D measures were exploratory endpoints in the STRIVE clinical trial and their analyses were not pre-specified therefore no statistical correction for multiplicity was undertaken. Accordingly, three sets of post-hoc analyses were subsequently conducted using trial-based data as described below. Analyses were undertaken using SAS/STAT® software, Versions 9.3 and 9.4 of the SAS System for Windows (Cary, NC, USA).

1. *Relationship between EQ-5D measures and ppFEV*_*1*_
  Mean values and 95 % confidence intervals for EQ-5D index and VAS scores were calculated using all observations across all study assessments and treatments and stratified by deciles of ppFEV_1_, and categories of lung dysfunction as described previously The proportions of patients with maximum value of EQ-5D index (=1) and VAS score (=100) respectively were calculated to assess ceiling effects.
2. *EQ-5D measures and ppFEV*_*1*_*among patients who experienced PEs*
  Data from patients who experienced at least one PE were included in this analysis. The periods prior and subsequent to the study defined PE start date were arbitrarily specified and grouped into pre-PE periods (>8 weeks, >4–8 weeks and > 1–4 weeks) and post-PE periods (>8 weeks, >4–8 weeks, > 1–4 weeks) respectively. The one-week pre- and post-PE start date constituted the reference category (i.e., “PE start period”). Mean (SD) duration of PEs was also calculated based on study defined PE start and end dates, overall and for patients for whom PEs required hospitalization and for those for whom they did not respectively (unadjusted).
  Observations were visually graphed and overlaid with local regressions (LOESS) to depict the EQ-5D index and VAS scores over time respectively. Mixed-effects models for repeated measures (MMRM) were employed to generate (least squares [LS]) mean values for each period controlling for baseline ppFEV_1_, age, sex, baseline body mass index (BMI), baseline sweat chloride, history of pancreatic insufficiency and baseline use of cycling antibiotics, and repeated observations. Models were also used to evaluate differences in EQ-5D index, VAS scores, and ppFEV_1_ between pre- and post-PE start periods and the reference period. A subgroup analysis was also conducted for patients for whom the absolute ppFEV_1_ value did not decline by 10 or more percentage points (from randomization to the closest ppFEV_1_ assessment) prior to the PE start date.
3. *Association of PEs, ppFEV*_*1*_*and EQ-5D measures*
  Data from all patients (those who experienced PEs during the clinical study and those who did not) were included in this analysis. For patients who experienced one or more PEs, EQ-5D index, VAS scores and ppFEV_1_ between PE start and end dates (inclusive of those dates) were considered related to a PE. Observations that did not occur during a PE window were not considered PE-related. Multivariate (MMRM) analyses were undertaken to examine the association of experiencing a PE (requiring and not requiring hospitalization), and ppFEV_1_ (with linear and quadratic terms) with the EQ-5D measures as dependent variables. As a sensitivity analysis, regression models were also developed using US, Europe, Netherlands, and Belgium EQ-5D index value sets (i.e., preference weights), [21–23] as well as for VAS scores that were transformed into a health-state utility value using a previously reported equation [24].

### Ethics

The STRIVE clinical trial (“A Phase 3, Randomized, Double-Blind, Placebo-Controlled, Parallel-Group Study to Evaluate the Efficacy and Safety of VX-770 in Subjects with Cystic Fibrosis and the G551D Mutation”) protocol was reviewed and approved by the institutional review board at each participating center, and each subject provided written informed consent or written or oral assent.

## Results

A total of 161 patients (age: mean [SD] 25.5 [9.5] years; baseline ppFEV_1_: mean [SD] 63.6 [16.4]) contributed 1,214 sets of observations (EQ-5D measures and ppFEV_1_) over 48 weeks. Of the 1,214 ppFEV_1_ assessments (no lung dysfunction [*n* = 157], mild [*n* = 149], moderate [*n* = 572], and severe [*n* = 66]), all but 12 occurred on the same day as the EQ-5D measurements. Over the course of the study, patients most frequently reported problems with pain/ discomfort (20.2 %), followed by anxiety/ depression (16.4 %), and usual activities (14.1 %). The EQ-5D index was at its ceiling (=1) for 67.5 % (no lung dysfunction: 80.9 %; mild: 73.3 %; moderate: 62.1 %; severe: 45.5 %) of observations whereas the EQ-5D VAS was at the ceiling (VAS = 100) for 5.6 % (no lung dysfunction: 15.9 %; mild: 6.9 %; moderate: 2.4 %; severe: 0.0 %) of observations.

A total of 146 PEs were experienced by 72 (44.7 % of total 161) patients, including 52 PEs (35.6 %) that required hospitalization. Mean (SD) duration was 30.0 (22.2) days for PEs requiring hospitalization and 20.6 (11.6) days for those not requiring hospitalization (11 PEs from 9 patients had missing PE end dates and were excluded). The baseline characteristics of patients who experienced a PE during the study and those who did not are summarized in Table 1. Patients who did not experience a PE during the study had on average a higher ppFEV_1_ at study initiation than those who experienced one or more PEs. Patients who experienced a PE requiring hospitalization were younger than those with a PE not requiring hospitalization and the mean ppFEV_1_ at study initiation did not differ between these two groups.

**Table 1:** Patient Characteristics at Baseline, by Pulmonary Exacerbations During Study

	No PE	Any PE	PE (No Hospitalization)	PE (Hospitalization)
	(*n* = 89)	(*n* = 72)	(*n* = 38)	(*n* = 34)
Age, Mean (SD)	25.5 (10.2)	25.4 (8.8)	28.3 (7.8)	22.0 (8.7)
Female, N(%)	41 (46.1 %)	43 (59.7 %)	20 (52.6 %)	23 (67.6 %)
ppFEV_1_, Mean (SD)	65.8 (15.8)	60.8 (16.9)	60.9 (16.3)	60.6 (17.7)
BMI, N(%)				
Normal Weight	75 (84.3 %)	57 (79.2 %)	28 (73.7 %)	29 (85.3 %)
Obese	3 (3.4 %)	2 (2.8 %)	2 (5.3 %)	0 (0 %)
Overweight	9 (10.1 %)	8 (11.1 %)	6 (15.8 %)	2 (5.9 %)
Underweight	2 (2.3 %)	5 (6.9 %)	2 (5.3 %)	3 (8.8 %)
Sweat chloride, Mean (SD)	100.5 (9.5)	99.9 (11.3)	100 (11.4)	99.8 (11.2)
History of pancreatic insufficiency, N(%)	81 (91.0 %)	68 (94.4 %)	35 (92.1 %)	33 (97.1 %)
Use of inhaled cycling antibiotic, N(%)	37 (41.6)	19 (26.4 %)	8 (21.1 %)	11 (32.4 %)

### EQ-5D measures and ppFEV_1_

**Fig. 1:**
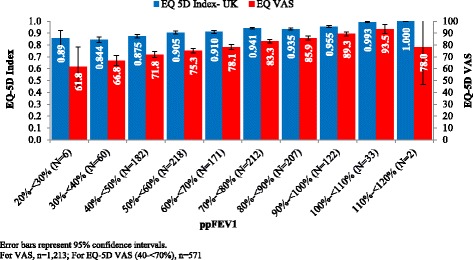
Mean EQ-5D Index and VAS Scores by ppFEV_1_ (Unadjusted)

Figure 1 shows EQ-5D index and VAS scores by ppFEV_1_ decile. VAS scores appeared to be more discriminating of CF lung disease severity than the EQ-5D index. Within MMRM models, the EQ-5D index (mean, [SE]) nominally decreased (worsened) with increasing severity of lung dysfunction (*P* = 0.070): no lung dysfunction: 0.931 (0.023); mild: 0.923 (0.021); moderate: 0.904 (0.018); severe: 0.870 (0.020). Mean (SE) VAS scores followed a similar trend and were significantly different (*P* < 0.001) across ppFEV_1_ categories: no lung dysfunction: 85.2 (2.0); mild: 82.3 (1.8); moderate: 76.8 (1.6); severe: 73.3 (1.8).

### EQ-5D measures and ppFEV_1_ by time since PE start

**Fig. 2:**
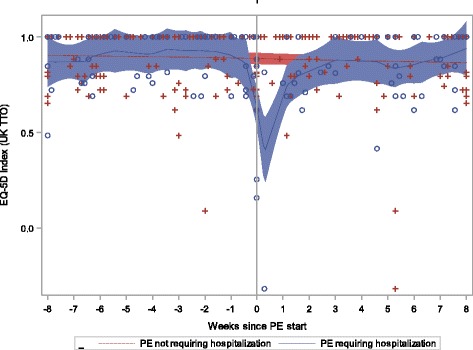
EQ-5D Index by Time since PE Start and Hospitalization

**Fig. 3:**
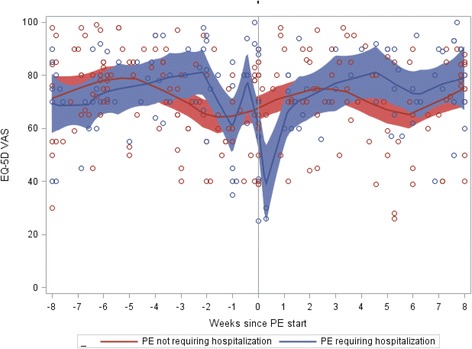
EQ-5D VAS Scores by Time since PE Start and Hospitalization

EQ-5D index and VAS scores over time in relation to PE start are shown in Figs. 2 and 3 respectively. Nominal trends for declining (worsening) values in the pre- PE start periods can be inferred from visual inspection of the data (both index and VAS scores) suggesting detrimental changes in health status and symptoms in advance of the study defined PE start date, followed by a trend for recovery.

For PEs that required hospitalization mean EQ-5D index, VAS scores and ppFEV_1_ values were lowest (worst) during the PE start period within MMRM (Table 2); mean values for the EQ-5D and ppFEV_1_ measures are suggestive of a trend towards recovery following the PE start period although findings did not always reach statistical significance (compared to the PE start period). A similar pattern is suggested by findings for PEs that did not require hospitalization, but overall, results did not achieve statistical significance. Findings from a subanalysis which removed patients who had a ppFEV_1_ decline of 10 or more percentage points at the observation nearest to the PE start date were consistent with similar detrimental impacts on the EQ-5D index (for PEs that required hospitalization), suggesting that PEs affect HRQL beyond the ppFEV_1_ decline alone (data not shown).

**Table 2:** EQ-5D index, VAS scores and ppFEV_1_ by time since Pulmonary Exacerbation Start

		EQ-5D Index		EQ-5D VAS		ppFEV_1_	
	n	LS Mean (SE)	*p*-value	LS Mean (SE)	*p*-value	LS Mean (SE)	*p*-value
PE Requiring Hospitalization							
>8 weeks prior	162	0.904 (0.020)	<0.001	72.8 (2.1)	0.015	60.9 (2.1)	0.043
>4–8 weeks prior	27	0.884 (0.030)	0.001	73.3 (3.1)	0.038	60.1 (2.5)	0.226
>1–4 weeks prior	20	0.905 (0.034)	<0.001	73.8 (3.5)	0.046	64.7 (2.6)	0.002
Within 1 week of start: reference group	23	0.760 (0.033)	-	65.3 (3.4)	-	57.6 (2.5)	-
>1–4 weeks post	15	0.841 (0.039)	0.084	74.5 (4.0)	0.053	65.5 (2.8)	0.002
>4–8 weeks post	29	0.847 (0.030)	0.022	72.7 (3.1)	0.052	63.4 (2.4)	0.004
>8 weeks post	121	0.856 (0.021)	0.002	72.0 (2.2)	0.034	62.7 (2.2)	0.003
PE Not Requiring Hospitalization							
>8 weeks prior	305^a^	0.883 (0.016)	0.787	72.1 (1.5)	0.021	59.3 (1.7)	0.029
>4–8 weeks prior	54	0.912 (0.024)	0.252	73.3 (1.9)	0.014	58.3 (1.8)	0.336
>1–4 weeks prior	36	0.876 (0.029)	0.988	72.5 (2.2)	0.071	60.6 (1.9)	0.015
Within 1 week of start: reference group	38	0.876 (0.027)	-	68.0 (2.1)	-	57.1 (1.9)	-
>1–4 weeks post	33	0.916 (0.029)	0.276	70.6 (2.2)	0.295	57.6 (1.9)	0.753
>4–8 weeks post	47	0.849 (0.025)	0.396	71.9 (2.0)	0.082	57.5 (1.8)	0.748
>8 weeks post	210	0.857 (0.017)	0.470	70.2 (1.5)	0.233	58.7 (1.7)	0.115

*Abbreviations*: *ppFEV*_*1*_ percent predicted forced expiratory volume in 1 s, *VAS* visual analog scale score, *LS* least-squares

^a^ *N* = 304 observations for ppFEV_1_

In examining Fig. 3, one might question the “peak” in VAS scores for patients who experienced a PE requiring hospitalization immediately prior to the PE start date. Of the 6 observations that occurred within the 3 days prior to exacerbation, 3 had VAS values that were > 90. Two of these three assessments were provided by patients with ppFEV_1_ < 70 % at the time of that visit; two additional observations, from different patients, also had a concurrent EQ-5D index < 1.

Mean ppFEV_1_ was significantly lower during the PE start period as compared to the preceding and subsequent periods in particular for PEs that required hospitalization. Findings were similar for PEs that did not require hospitalization but did not always reach statistical significance when compared to the PE start period.

Between PE start and end dates, patients were most likely to report problems for usual activities (53.6 % of observations), pain/discomfort (39.3 %) and mobility (32.1 %) for PEs that required hospitalization. For PEs not requiring hospitalization, the trend was similar but fewer patients reported problems: 35.7 % for usual activities, 28.6 % for pain/discomfort, and 16.7 % for both anxiety and depression and mobility. Problems were reported on at least one dimension of the EQ-5D for 52.4 % of observations for hospitalized and non-hospitalized PEs and for 31.5 % of observations that did not occur during a PE.

### Association of PEs, ppFEV_1_ and EQ-5D measures

In multivariate analyses, PE and low ppFEV_1_ values were significantly (*P* <0.05) associated with a lower (worse) EQ-5D index (Table 3). When PEs were stratified by hospitalization status, experiencing a PE requiring hospitalization and low ppFEV_1_ at the observation time were significant (*p* < 0.05) predictors of EQ-5D index. PEs not requiring hospitalization had a negligible positive effect (*P* = 0.965). In VAS models, PEs, PEs requiring hospitalization, and low ppFEV_1_ were associated with lower (worse) VAS scores (Table 3). Interestingly PEs not requiring hospitalization had a greater (negative) effect on VAS scores as compared to PEs requiring hospitalization. Conclusions did not change when applying US, Belgium, Netherlands, European algorithms reflecting country-specific preference weights and the VAS transformed algorithm (Additional file 1).

**Table 3:** Association of ppFEV_1_ and Pulmonary Exacerbations with EQ-5D Index and VAS

Parameter	Model 1: ppFEV_1_ Only	Model 2: ppFEV_1_ + Any PE	Model 3: ppFEV_1_ + PE Type
EQ-5D Index			
Intercept	0.670 (0.068)**	0.678 (0.067)**	0.686 (0.067)**
ppFEV_1_	0.580 (0.193)**	0.561 (0.193)*	0.535 (0.193)*
ppFEV_1_ squared	−0.305 (0.135)**	−0.294 (0.135)*	−0.274 (0.135)*
Any PE	–	−0.026 (0.013)*	–
PE (Hospitalization)	–	–	−0.070 (0.020)*
PE (No Hospitalization)	–	–	0.001 (0.016)
EQ-5D VAS			
Intercept	57.79 (2.38)**	58.46 (2.36)**	58.48 (2.36)**
ppFEV_1_	32.14 (3.23)**	31.52 (3.21)**	31.49 (3.21)**
Any PE	–	−4.4 (1.08)**	–
PE (Hospitalization)	–	–	−3.82 (1.69)*
PE (No Hospitalization)	–	–	−4.75 (1.34)**

*Abbreviations*: *PE* pulmonary exacerbation, *ppFEV*_*1*_ percent predicted forced expiratory volume in 1 second, *VAS* visual analog scale

*p < 0.05 **p < 0.001

## Discussion

This analysis provides the first assessment of the impact of both lung function and PEs on a generic HRQL measure in patients with CF. In post-hoc analyses of data from a clinical study of patients with CF (≥12 years of age and a *G551D-*CFTR mutation), PEs, primarily those requiring hospitalization, were associated with lower HRQL as measured by the EQ-5D index. Mean EQ-5D index was lowest (worst) within one week (before or after PE start) for PEs requiring hospitalization. Similar findings were observed for ratings of health status as measured by VAS scores for all PEs regardless of whether they required hospitalization. Lung function as measured by ppFEV_1_ was also on average lowest (worst) within the one-week period before or after the study defined PE start date regardless of whether PEs required hospitalization or not. In multivariate analyses, experience of any PEs, PEs requiring hospitalization and low ppFEV_1_ were identified as independent negative predictors of EQ-5D index and VAS scores.

Our study findings are consistent with those reported by others [5, 6]. A prior meta-analysis has identified PEs and ppFEV_1_ as predictors of disease-specific HRQL and symptoms as measured by Cystic Fibrosis Questionnaire-Revised (CFQ-R) scores [5]. In cross-sectional analyses of data from pediatric and adult patients with CF treated in a US Midwestern CF center, PEs were reported to have a profound negative impact on physical and psychosocial HRQL using the generic Short-Form (SF)-36 and the Child Health Questionnaire; the impact of ppFEV_1_ was reported to be relatively small compared to the impact of PEs [8]. In another study conducted in five UK CF centers, adolescent patients with CF and chronic *Pseudomona aeruginosa* infection who experienced PEs that required hospitalization reported poorer HRQL using the EQ-5D index as well as worse VAS scores compared to those with chronic infection and milder PEs (i.e., not requiring hospitalization) [6].

In our analyses, ceiling effects were high, particularly for the EQ-5D index and in patients with no lung dysfunction or mild lung dysfunction as well as among those with less severe disease (i.e., patients who did not experience PEs). Mixed results have been reported by others regarding the association between physiologic measures of pulmonary disease and CF, particularly ppFEV_1_, nutritional indices and HRQL [8, 25–27]. Patients with the most severe disease as measured by pulmonary function measures and weight for height have been reported to rate their CF as “above/well above average” compared to other patients with CF whereas physician’s ratings of disease severity were positively correlated with clinical findings [28]. Yi et al.’s study of adolescents with CF that employed preference elicitation methods (e.g., time tradeoff, standard gamble) found that HRQL was poorly associated with lung function [7]. In our subgroup analysis, HRQL impacts of PEs requiring hospitalization were still apparent after exclusion of patients who did not experience a decline in absolute ppFEV_1_ of 10 percentage points or higher prior to the PE start date.

In summary, our results are supportive of prior suggestions that “the powerful association of HRQL with exacerbations, and the weaker association with FEV_1_ percent predicted may imply that for patients with CF and their families, HRQL may have less to do with how severe one’s underlying disease is, and more to do with the disruptive effect of exacerbations.” [8]. While this may be more strongly observed for disease specific measures, there is an important role for including and assessing the impact of disease on generic measures, which are frequently used for cross-condition comparisons. The STRIVE trial appropriately included the EQ-5D as a generic measure of HRQL and the Cystic Fibrosis Questionnaire- Revised which has been reported elsewhere [29].

It should be noted that the EQ-5D index at the time of study initiation was high (mean ≈ 0.93) leaving little room for improvement with study treatments. There are multiple possible explanations for these high values. Among others, STRIVE study criteria excluded patients with a history of any illness or condition that, in the opinion of the investigator, could confound the results of the study or pose an additional risk in administering study drug to the patients, acute respiratory illness or PE within four weeks of baseline, those with colonization with selected microorganisms, and patients with any “non-CF-related” illness within 2 prior weeks. High scores have been also reported at the time of study initiation in the TIGER clinical trial (Health State Utilities Index [HUI] = 0.90, and 0.83 using a feeling thermometer similar to the VAS) [15]. High values may also be explained by patients’ adaptation and coping mechanisms leading to acceptance of their chronic condition. From the physician’s point of view, patients with CF and their close companions may underestimate the severity of their disease and overestimate self-care, and such perceptions often remain constant over time even if the patient’s health is clinically deteriorating [28]. Denial of physical symptoms as a protective means for psychosocial adaptation and emotional resiliency are suggested strategies for living in the present used by patients with chronic disease which may limit consideration of the full impact of their condition when evaluating health on a given day [15, 30–33].

While both EQ-5D and VAS are generic measures, they provide complementary information; the use of the EQ-5D index alone (a generic HQRL measure) may limit characterization of disease burden and health gains in patients with CF. The EQ-5D index is however, a necessary generic HRQL (“health-state utility”) metric that is typically required in cost-effectiveness evaluations of new therapies [34]. In our analysis, the EQ-5D VAS measure showed greater ability to discriminate disease severity (as measured by ppFEV_1_ and PEs respectively) than the EQ-5D index. This broader range of response on the VAS measure, as compared to the EQ-5D index, which has also been reported by others [6], may point to health constructs or dimensions that may not be fully captured by the EQ-5D questionnaire. Some dimensions of the EQ-5D, particularly self-care, are less likely to be impacted by CF; pain, discomfort, anxiety and depression have been reported to be most affected in previous studies [16, 35, 36] but others may not be well represented by this generic HRQL measure. Use of disease-specific measures (e.g., CFQ-R) alongside generic instruments, per guidance, [1] should provide complementary assessment of patient-reported symptoms and HRQL.

The current analysis does have a number of limitations. EQ-5D measures were not assessed at the time of PE start as this was not part of the original study design. Sample size was small for ppFEV_1_ category <40 % and for some of the PE-related analytical windows employed in our analyses. Patients in clinical practice may differ from those participating in the STRIVE clinical trial and caution should be used in generalization of study findings. Also, the STRIVE clinical study included only patients with CF and the *G551D-*CFTR mutation. Finally, the EQ-5D was designed for use in populations 18 years of age or older, [19] whereas this study included adolescent patients as young as 12 years of age. However, it should be noted that some previous research has indicated that as long as the language and concepts used within the instrument are understood, the EQ-5D may be used in adolescents (12–18 years) with adequate functioning ([37] as cited in [38]).

## Conclusion

In summary, in a clinical study of patients with CF (≥12 years of age and a *G551D*-CFTR mutation), PEs, primarily those requiring hospitalization, were associated with low EQ-5D index and VAS scores. The impact of ppFEV_1_ was relatively smaller. Reducing PEs, in particular those requiring hospitalization, is likely to improve HRQL among these patients.

### Availability of data and materials

Supplemental data and materials are available upon request.

### Ethics approval and consent to participate

This was a secondary analysis of the STRIVE clinical trial data. Please see original trial [18] for information on ethics approval.

## Additional file

Additional file 1: Mixed model parameter estimates in models predicting EQ-5D utility values using US, European and Belgium EQ-5D algorithms and VAS-transformed utilities. (DOCX 15 kb)

## Abbreviations

BMI: body mass index
CF: cystic fibrosis
HRQL: health-related quality of life
LOESS: non-parametric local regression smoothing
PE: pulmonary exacerbation
ppFEV_1_: percent predicted forced expiratory volume in 1 second
VAS: visual analog scale
