# Supplementary material for: Impact of pulmonary exacerbations and lung function on generic health-related quality of life in patients with cystic fibrosis
Source: Health Qual Life Outcomes. 2016 Apr 21;14:63. doi: 10.1186/s12955-016-0465-z (PMC4839094; doi:10.1186/s12955-016-0465-z)
Supplement: Additional file 1: — Mixed model parameter estimates in models predicting EQ-5D utility values using US, European and Belgium EQ-5D algorithms and VAS-transformed utilities. (DOCX 15 kb) [file 12955_2016_465_MOESM1_ESM.docx]

**Appendix for possible web publication**

**Mixed model parameter estimates in models predicting EQ-5D and utility measures**

1. **US algorithm models**

| Parameter | Model 1:  ppFEV_1_ Only | Model 2:  ppFEV_1_ + Any PE | Model 3:  ppFEV_1_ + PE Type |
| --- | --- | --- | --- |
| Intercept | 0.7523 (0.0502)** | 0.7601 (0.05020)** | 0.7658 (0.05013)** |
| ppFEV_1_ | 0.4181 (0.1441)* | 0.4019 (0.1440)* | 0.3830 (0.1438)* |
| ppFEV_1_ squared | -0.2154 (0.1010)* | -0.2062 (0.1009)* | -0.1917 (0.1008) |
| Any PE | – | -0.02312 (0.009691)* | – |
| PE (Hospitalization) | – | – | -0.05429 (0.01513)** |
| PE (No Hospitalization) | – | – | -0.00440 (0.01192) |

*** p<0.05 ** p<0.001**

1. **European algorithm models**

| Parameter | Model 1:  ppFEV_1_ Only | Model 2:  ppFEV_1_ + Any PE | Model 3:  ppFEV_1_ + PE Type |
| --- | --- | --- | --- |
| Intercept | 0.6843 (0.06502)** | 0.6932 (0.6505)** | 0.6999 (0.06499)** |
| ppFEV_1_ | 0.5132 (0.1862)* | 0.4946 (0.1861)* | 0.4726 (0.1860)* |
| ppFEV_1_ squared | -0.2586 (0.1303)* | -0.2480 (0.1302) | -0.2312 (0.1302) |
| Any PE | – | -0.02632 (0.01245)* | – |
| PE (Hospitalization) | – | – | -0.06213 (0.01945)* |
| PE (No Hospitalization) | – | – | -0.00484 (0.01532) |

*** p<0.05 ** p<0.001**

1. **Belgium algorithm models**

| Parameter | Model 1:  ppFEV_1_ Only | Model 2:  ppFEV_1_ + Any PE | Model 3:  ppFEV_1_ + PE Type |
| --- | --- | --- | --- |
| Intercept | 0.6493 (0.07179)** | 0.6605 (0.07178)** | 0.6683 (0.07170)** |
| ppFEV_1_ | 0.5700 (0.2057)* | 0.5464 (0.2055)* | 0.5210 (0.2053)* |
| ppFEV_1_ squared | -0.2867 (0.1440)* | -0.2733 (0.1438) | -0.2539 (0.1437) |
| Any PE | – | -0.03338 (0.01376)* | – |
| PE (Hospitalization) | – | – | -0.07500 (0.02149)** |
| PE (No Hospitalization) | – | – | -0.00841 (0.01693) |

*** p<0.05 ** p<0.001**

1. **Netherlands algorithm models**

| Parameter | Model 1:  ppFEV_1_ Only | Model 2:  ppFEV_1_ + Any PE | Model 3:  ppFEV_1_ + PE Type |
| --- | --- | --- | --- |
| Intercept | 0.7034 (0.06124)** | 0.7112 (0.06128)** | 0.7187 (0.06117)** |
| ppFEV_1_ | 0.5115 (0.1757)* | 0.4952 (0.1757)* | 0.4705 (0.1754)* |
| ppFEV_1_ squared | -0.2659 (0.1231)* | -0.2566 (0.1230)* | -0.2378 (0.1228) |
| Any PE | – | -0.02328 (0.01180)* | – |
| PE (Hospitalization) | – | – | -0.06374 (0.01842)* |
| PE (No Hospitalization) | – | – | 0.001007 (0.01451) |

*** p<0.05 ** p<0.001**

1. **VAS-Transformed Algorithm Models**

| Parameter | Model 1:  ppFEV_1_ Only | Model 2:  ppFEV_1_ + Any PE | Model 3:  ppFEV_1_ + PE Type |
| --- | --- | --- | --- |
| Intercept | 0.6546 (0.04579)** | 0.6654 (0.04560)** | 0.6648 (0.04566)** |
| ppFEV_1_ | 0.5218 (0.1316)** | 0.4993 (0.1310)** | 0.5013 (0.1312)** |
| ppFEV_1_ squared | -0.2177 (0.9226) | -0.2049 (0.09181)* | -0.2064 (0.09198)* |
| Any PE | – | -0.03295 (0.00885)** | – |
| PE (Hospitalization) | – | – | -0.02972 (0.01386)* |
| PE (No Hospitalization) | – | – | -0.03490 (0.01092)* |

*** p<0.05 ** p<0.001**
